# Theory of Mind Variability in Schizophrenia: A Neurodevelopmental Perspective through Neurological Soft Signs and Premorbid Adjustment

**DOI:** 10.1192/j.eurpsy.2026.10527

**Published:** 2026-07-31

**Authors:** M. Giralt Lopez, S. Miret, S. Campanera, M. Moreira, A. Sotero-Moreno, N. Hostalet, L. Lázaro, M.-O. Krebs, L. Fañanás, M. Fatjó-Vilas

**Affiliations:** 1 Departament de Psiquiatria i Medicina Legal, Universitat Autònoma de Barcelona, Bellaterra; 2 Germans Trias i Pujol Research Institute; 3 Child and Adolescent Psychiatry, Hospital Universitari Germans Trias i Pujol, Badalona; 4 Psychiatry, Hospital Universitari Santa Maria, Lleida; 5 Centro de Investigación Biomédica en Red de Salud Mental (CIBERSAM), Instituto de Salud Carlos III, Madrid; 6 FIDMAG Germanes Hospitalàries Research Foundation; 7 Departament de Biologia Evolutiva, Ecologia i Ciències Ambientals, Facultat de Biologia, Universitat de Barcelona; 8 Servei de Psiquiatria i Psicologia Infantil i Juvenil, Hospital Clínic, IDIBAPS; 9 Departament de Medicina, Facultat de Medicina i Ciències de la Salut, Universitat de Barcelona, Barcelona, Spain; 10 Institute of Psychiatry and Neuroscience of Paris (INSERM U1266), GHU-Paris Psychiatrie et Neurosciences, Université Paris Cité, Paris, France

## Abstract

**Introduction:**

Theory of Mind (ToM) is impaired in individuals with schizophrenia (SZ). Given the neurodevelopmental nature of both social cognition and SZ, variations in ToM abilities likely originate early in life. Thus, indirect markers of altered neurodevelopment, such as neurological soft signs (NSS) and premorbid adjustment (PA), may help explain these differences.

**Objectives:**

To compare schizophrenia patients, their siblings, and controls on ToM and neurodevelopmental deviation, assessed through neurological soft signs (NSS) and premorbid adjustment (PA), and determine whether these markers explain ToM variability.

**Methods:**

The study included 38 patients with schizophrenia-spectrum disorder (SSD), 26 healthy siblings and 47 controls. ToM was assessed using the Hinting Task (HT). NSS were evaluated with the Neurological Evaluation Scale (NES) and PA with the Premorbid Adjustment Scale (PAS), yielding Social and Academic scores. Intelligence Quotient (IQ) was estimated two subtests of the WAIS-III and Family History (FH) through the Family Interview for Genetic Studies (FIGS).

**Results:**

First, patients presented more deficits in two subscales of the NES (motor coordination and sequencing of complex motor acts) than siblings and controls, with siblings performing intermediate in the sequencing subscale (linear mixed models, adjusted for age, sex and IQ). Patients showed worse social PA than siblings during childhood and late adolescence. Second, patients showed poorer HT performance than siblings and controls, but the neurodevelopmental markers did not modulate such differences. Third, within each group, neurodevelopmental vulnerability markers were not associated with ToM performance (linear regression analyses adjusted for age, sex, IQ and FH).

**Conclusions:**

In our sample, while patients showed more evidence of neurodevelopmental deviances than siblings and controls, such differences did not contribute to ToM variability, suggesting that they are partially independent and potentially influenced by distinct neurodevelopmental mechanisms.

Acknowledgements: ISCIII project-PI20/01002 and PI23/01262, co-funded by ERDF/ESF.

**Disclosure of Interest:**

None Declared

